# Excited State Lifetimes of Sulfur-Substituted DNA and RNA Monomers Probed Using the Femtosecond Fluorescence Up-Conversion Technique

**DOI:** 10.3390/molecules25030584

**Published:** 2020-01-29

**Authors:** Matthew M. Brister, Thomas Gustavsson, Carlos E. Crespo-Hernández

**Affiliations:** 1 Department of Chemistry, Case Western Reserve University, 10900 Euclid Ave., Cleveland, OH 44106, USA; mmbrister@lbl.gov; 2 LIDYL, CEA, CNRS, Université Paris-Saclay, F-91191 Gif-sur-Yvette, France

**Keywords:** fluorescence lifetimes, excited-state dynamics, thiobases, prodrugs, DNA and RNA derivatives

## Abstract

Sulfur-substituted DNA and RNA nucleobase derivatives (a.k.a., thiobases) are an important family of biomolecules. They are used as prodrugs and as chemotherapeutic agents in medical settings, and as photocrosslinker molecules in structural-biology applications. Remarkably, excitation of thiobases with ultraviolet to near-visible light results in the population of long-lived and reactive triplet states on a time scale of hundreds of femtoseconds and with near-unity yields. This efficient nonradiative decay pathway explains the vanishingly small fluorescence yields reported for the thiobases and the scarcity of fluorescence lifetimes in the literature. In this study, we report fluorescence lifetimes for twelve thiobase derivatives, both in aqueous solution at physiological pH and in acetonitrile. Excitation is performed at 267 and 362 nm, while fluorescence emission is detected at 380, 425, 450, 525, or 532 nm. All the investigated thiobases reveal fluorescence lifetimes that decay in a few hundreds of femtoseconds and with magnitudes that depend and are sensitive to the position and degree of sulfur-atom substitution and on the solvent environment. Interestingly, however, three thiopyrimidine derivatives (i.e., 2-thiocytidine, 2-thiouridine, and 4-thiothymidine) also exhibit a small amplitude fluorescence component of a few picoseconds in aqueous solution. Furthermore, the *N*-glycosylation of thiobases to form DNA or RNA nucleoside analogues is demonstrated as affecting their fluorescence lifetimes. In aqueous solution, the fluorescence decay signals exciting at 267 nm are equal or slower than those collected exciting at 362 nm. In acetonitrile, however, the fluorescence decay signals recorded upon 267 nm excitation are, in all cases, faster than those measured exciting at 362 nm. A comparison to the literature values show that, while both the DNA and RNA nucleobase and thiobase derivatives exhibit sub-picosecond fluorescence lifetimes, the ^1^ππ* excited-state population in the nucleobase monomers primarily decay back to the ground state, whereas it predominantly populates long-lived and reactive triplet states in thiobase monomers.

## 1. Introduction

Sulfur-substituted nucleobase monomers (a.k.a., thiobases) belong to a family of molecules known as prodrugs, which are commonly prescribed as immunosuppressants for organ transplant patients [1] and as maintenance therapy compounds for acute lymphoblastic leukemia, inflammatory bowel disease [2], and gliomas [3,4]. In addition, they are widely used as cytotoxic agents of clinical relevance [5,6,7] and in photo-chemotherapeutic applications to treat skin cancer cells [8,9,10,11] and other skin disorders [12], superficial tumors [13,14], and bladder cancer [15].

Despite the clinical and medical benefits of thiobases as chemotherapeutic prodrugs, they can be phototoxic. Laboratory experiments have shown that human cells can metabolize and incorporate 6-thioguanine, 6-mercaptopurine, and azathioprine prodrugs into their native DNA as the 6-thioguanosine metabolite [1,13,16,17,18,19,20,21,22]. This incorporation results in DNA mutations and cell death upon exposure to low doses of ultraviolet-A (UVA) irradiation that would otherwise be nonlethal [13]. Equally important is the fact that the skin of patients treated with these drugs is sensitive to UVA radiation and long-term treatment of patients results in substantial increases in the incidence of sunlight-induced skin cancer [1,16,23]. A key reason for this increase in the incidence of skin cancer is that UVA irradiation of DNA containing thiobases results in DNA oxidation, breakage, and crosslinking [1,7,13,16,24]. In addition, it has been proposed that photosensitized reactions play an important role in generating these mutagenic and cytotoxic DNA lesions [1,12,16]. While damage to DNA is manifested as structural modifications to the DNA itself, a complete understanding of the photochemical pathways leading to DNA damage requires describing both the radiative and nonradiative electronic relaxation pathways of these thiobases upon the absorption of UV radiation.

For this purpose, the excited-state dynamics of thiobases have recently received increased attention [25,26,27,28] because these compounds exhibit unique photochemical properties that make them promising for photodynamic and structural–biology applications [10,25,29,30,31,32,33,34]. Both experimental and theoretical calculations have shown that ultraviolet (either UVA, UVB, or UVC) excitation of thiobases results in ultrafast, intersystem crossing to the triplet manifold, which enables a near unity population of the triplet state, and allows these biomolecules to function as effective photosensitizers. The triplet state of the thiobases can also transfer their energy to molecular oxygen, leading to the efficient generation of singlet oxygen and other reactive oxygen species in solution [26]. 

The emphasis of the present study is to report fluorescence lifetimes for a wide-ranging set of DNA and RNA thiobase monomers at two different excitation wavelengths in an effort to develop a comprehensive understanding of their electronic relaxation mechanisms. While femtosecond broadband transient absorption spectroscopy has been successfully used to investigate the nonradiative decay pathways in a large number of thiobases [26], femtosecond fluorescence spectroscopy has only been applied to 4-thiothymidine in aqueous solution [25]. 

**Scheme 1:**
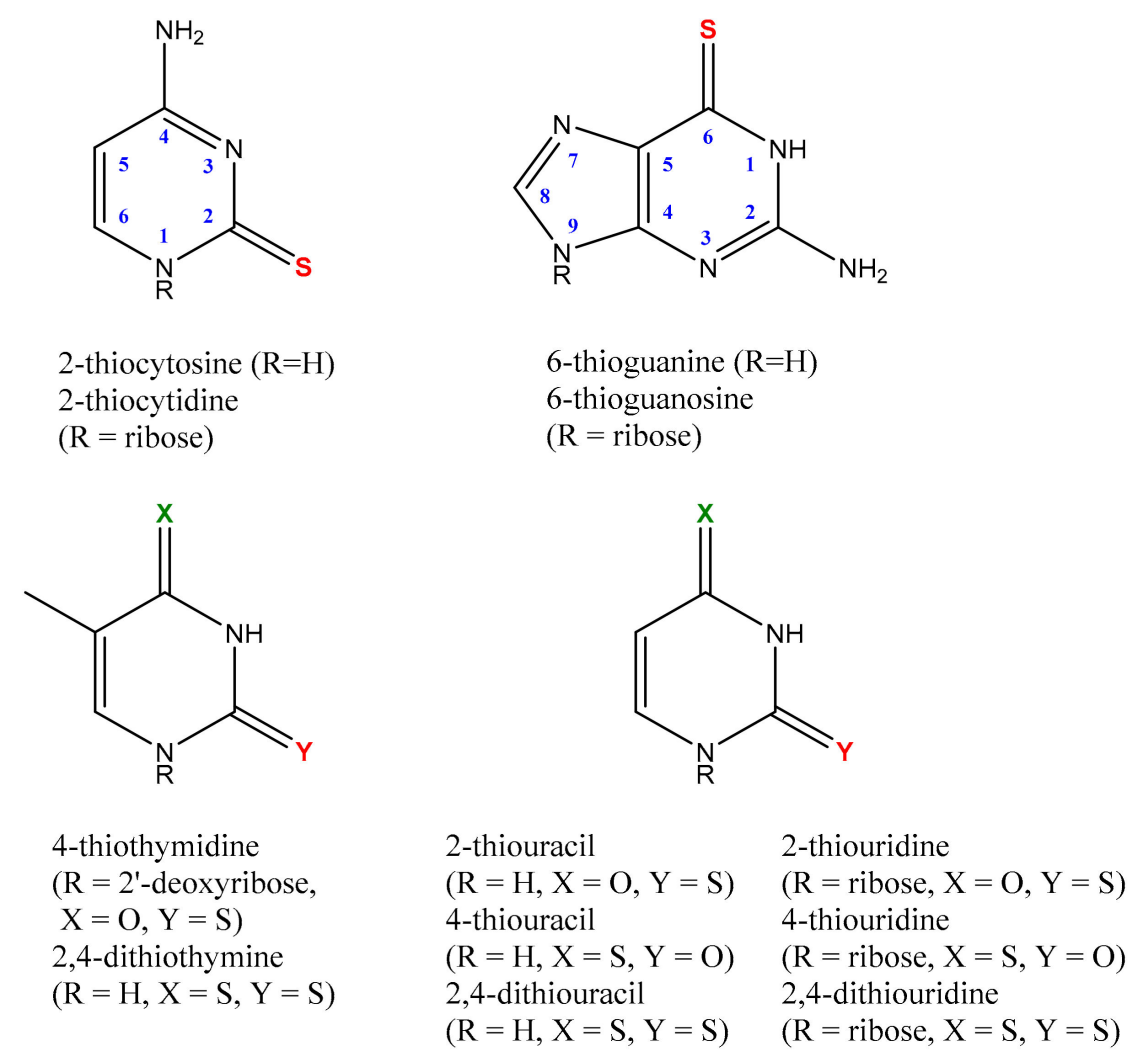
Structures and common ring numbering of the thiobases.

In this study, two thiopurines, 6-thioguanine (6tGua) and 6-thioguanosine (6tGuo), and ten thiopyrimidines, 2-thiocytosine (2tCyt), 2-thiocytidine (2tCyd), 4-thiothymidine (4tThd), 2-thiouracil (2tUra), 2-thiouridine (2tUrd), 4-thiouracil (4tUra), 4-thiouridine (4tUrd), 2,4-dithiothymine (2,4dtThy), 2,4-dithiouracil (2,4dtUra), and 2,4-dithiouridine (2,4dtUrd), are investigated using the fluorescence up-conversion technique, both in phosphate buffer saline (PBS) solution at pH 7.4 and in acetonitrile. The structures and standard ring numbering of the thiobases investigated in this work are shown in Scheme 1. 

We aim to reveal whether the position and number of sulfur-atom substitutions, the presence of a sugar, and the solvent environment (protic versus aprotic) affect the fluorescence lifetimes of these thiobase derivatives. We also investigate the effect of using two different excitation wavelengths on the fluorescence lifetimes of a sub-set of thiobases. This mechanistic information is necessary to complement recent transient absorption measurements that directly probe the intersystem crossing dynamics to the triplet manifold in many of the thiobase derivatives [25,26,28], and for ongoing efforts to understand the excited-state dynamics and photochemistry of thiobases when incorporated in DNA and RNA oligonucleotides.

## 2. Results

**Figure 1:**
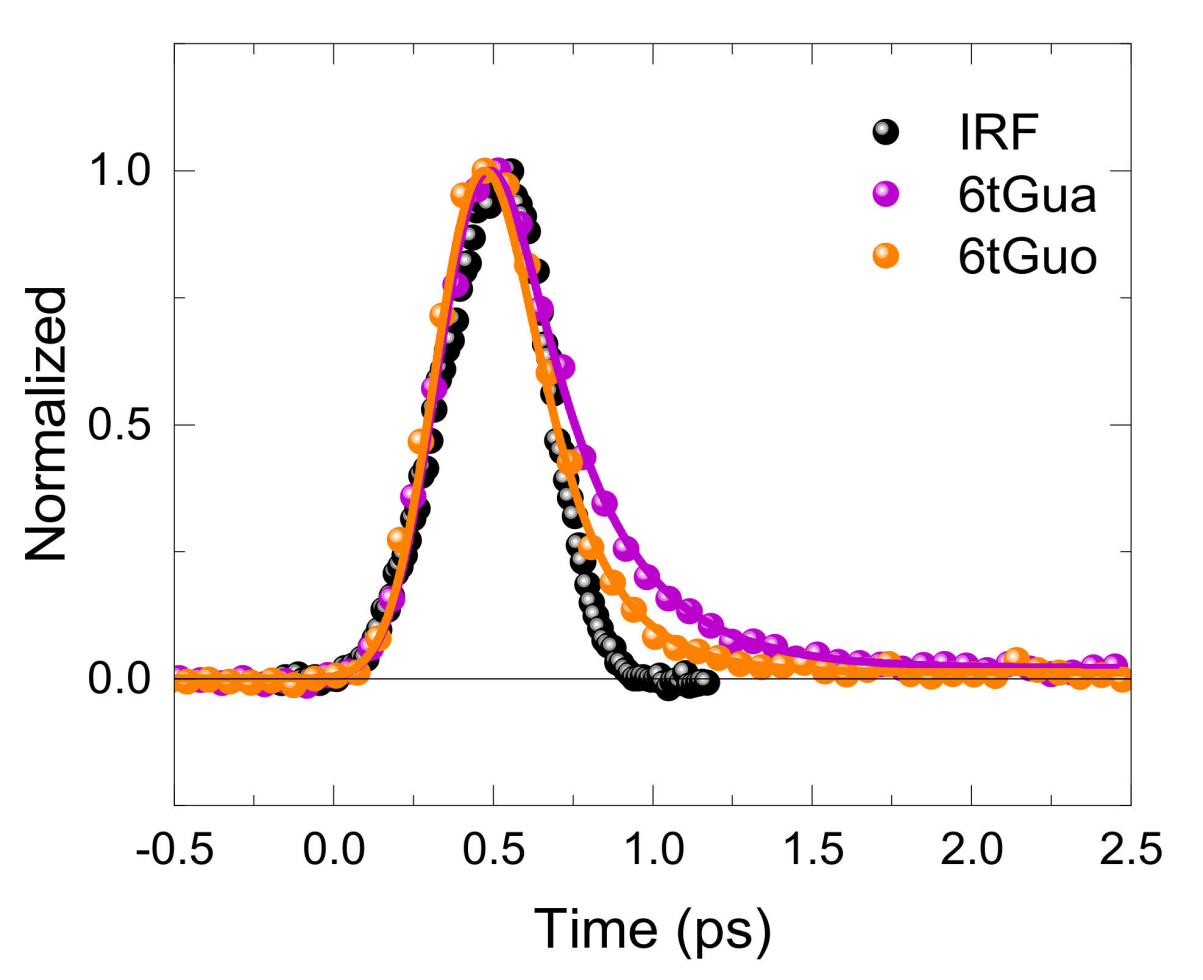
Fluorescence up conversion signals of 6tGua and 6tGuo monomers at the emission wavelength of 380 nm upon excitation at 267 nm in PBS at pH 7.4.

**Figure 2:**
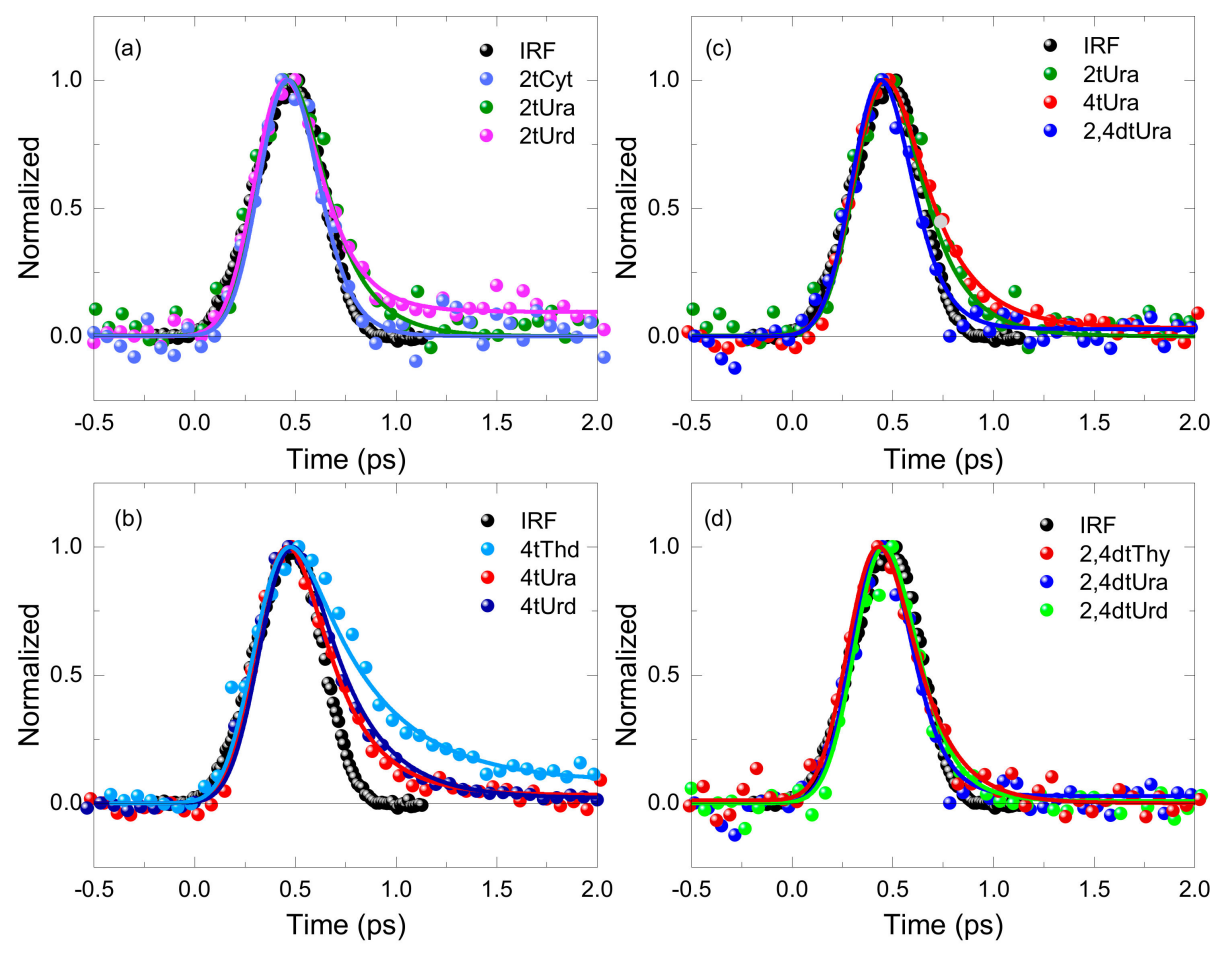
Fluorescence up conversion data for (**a**) 2-thiopyrimidines, (**b**) 4-thiopyrimidines, (**c**) 2-thiouracil, 4-thiouracil, and 2,4-dithiouracil, and (**d**) 2,4-dithiopyrimidines in PBS at pH 7.4 at the emission wavelength of 380 nm upon excitation of the thiobase monomers at 267 nm.

**Figure 3:**
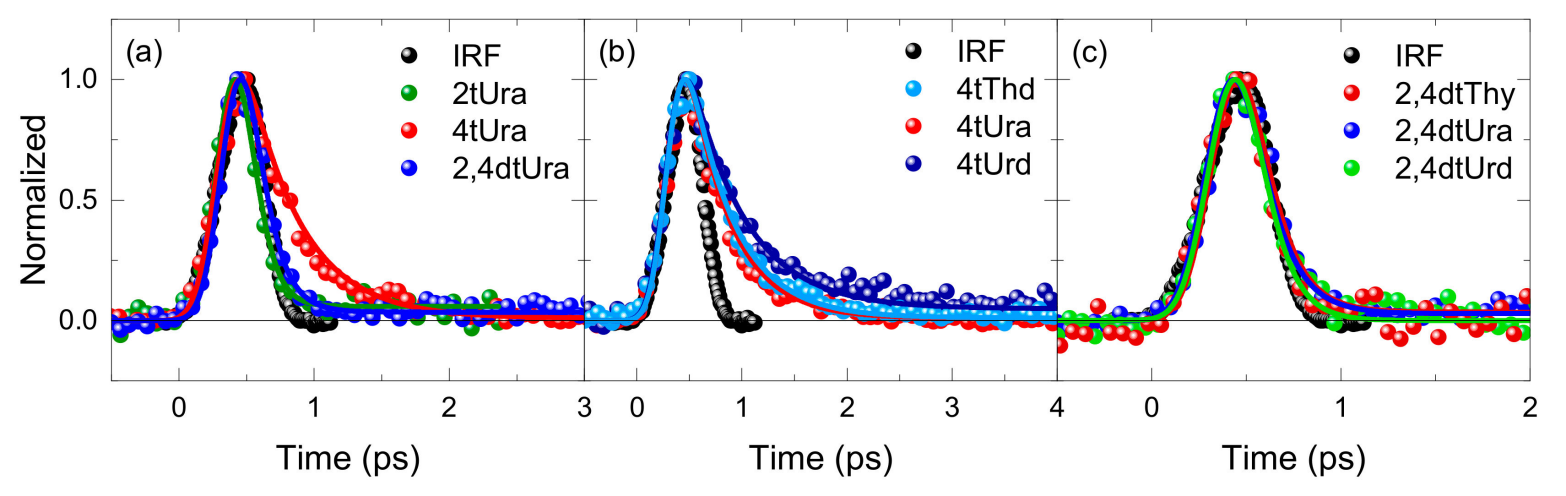
Fluorescence up conversion data for (**a**) 2-thiouracil, 4-thiouracil, and 2,4-dithiouracil, (**b**) 4-thiopyrimidines, and (**c**) 2,4-dithiopyrimidines in acetonitrile at the emission wavelength of 380 nm upon excitation of the thiobase monomers at 267 nm.

**Figure 4:**
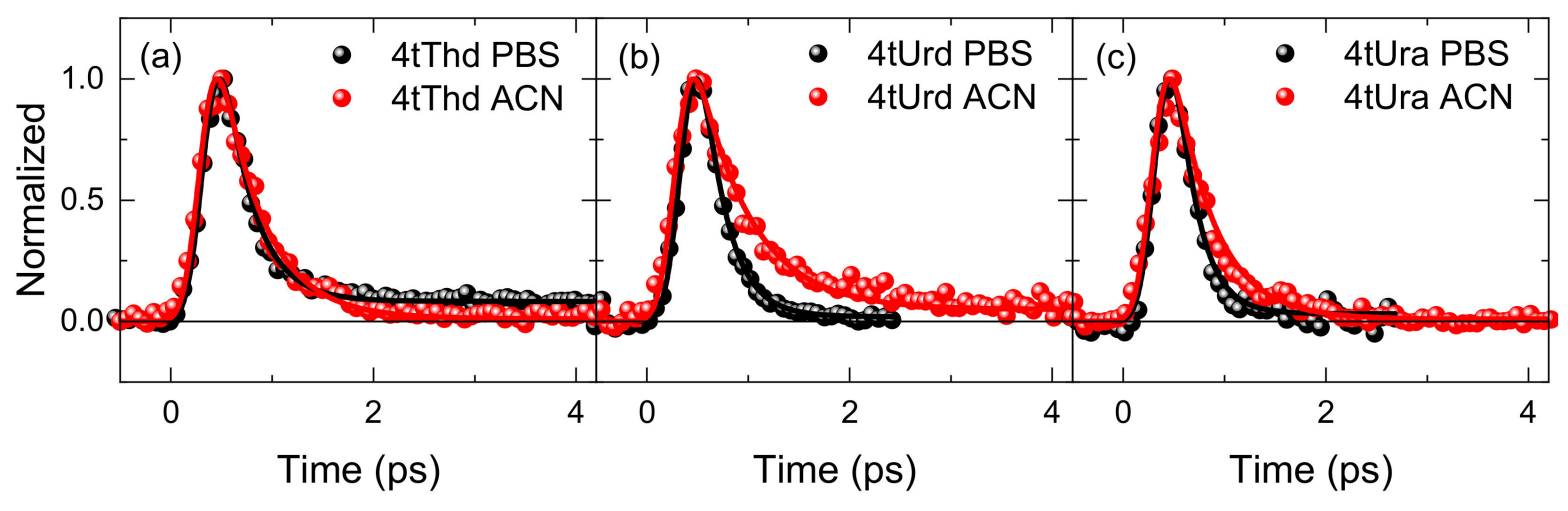
Effect of solvent environment on the fluorescence decay of (**a**) 4tThd, (**b**) 4tUrd, and (**c**) 4tUra. Fluorescence decay lifetimes were measured in PBS at pH 7.4 and in acetonitrile (ACN), exciting at 267 nm and a detecting emission at 380 nm.

The absorption and emission spectra of the thiobases are reported in Figures S1–S3, in both PBS and acetonitrile. All the fluorescence decay signals reported in Figure 1, Figure 2, Figure 3 and Figure 4 were collected using the fluorescence up-conversion technique, detecting the thiobases fluorescence emission at 380 nm upon excitation at 267 nm. Fluorescence decay signals for selected thiobases exciting at 362 nm and detecting the emissions at 425, 450, 525, and 532 nm were also collected in PBS or in acetonitrile, and are reported in Figures S4 and S5 (see also Table S1).

Figure 1 shows the normalized isotropic fluorescence decay signals of 6tGua and 6tGuo in PBS at pH 7.4. In particular, this figure displays the impact of the N-glycosidic bond on the fluorescence lifetime of the thiopurine bases. Note that Figure 1, as well as Figure 2; Figure 3 below, also show the Raman signal of the PBS aqueous solvent, which was used to estimate the instrument response function (IRF) of the setup.

Figure 2 depicts the isotropic fluorescence decay measurements of selected groups of thiopyrimidine monomers in PBS at pH 7.4. In panels (a) and (b) of Figure 2, we display the fluorescence decay signals for 2-thiopyrimidine and 4-thiopyrimidine derivatives, as well as the effect of N-glycosidic bond formation for the case of 2tUra and 4tUra, respectively. Figure 2c compares the effect that the position and degree of the thionation have on the fluorescence decay of the thiouracil derivatives, whereas Figure 2d compares the fluorescence decay between 2,4dtUra and 2,4dtThy, as well as the effect of glycosylation for the case of 2,4dtUra. 

Figure 3 presents the isotropic fluorescence decays of selected groups of thiopyrimidine derivatives using acetonitrile as the solvent. Figure 3a compares the effect that the position and degree of thionation have on the fluorescence decay of the thiouracil derivatives, while Figure 3b depicts the fluorescence decay of the 4-thiopyrimidine derivatives. Figure 3c shows the fluorescence decay signals of the double-substituted thiopyrimidine derivatives and the effect of the N-glycosidic bond formation for the case of 2,4dtUra. 

Finally, Figure 4 compares the fluorescence decay signals of 4tThd, 4tUrd, and 4tUra, both in aqueous solution at pH 7.4 and in acetonitrile. Table 1 and Table 2 collect the fluorescence lifetimes extracted after deconvolution of a 300 fs IRF from the fluorescence decay signals for all the thiobase derivatives investigated at 267 or 362 nm excitation wavelengths in PBS and in acetonitrile solutions, respectively. In both tables, the fluorescence lifetimes are further compared side-by-side with the ultrafast lifetimes reported in the literature, which were measured using femtosecond transient absorption experiments in the same solvents and at similar excitation wavelengths.

## 3. Discussion

The fluorescence up-conversion measurements presented in this study provide complementary measurements of the excited state lifetimes of twelve thiobase derivatives shown in Scheme 1. These fluorescence lifetimes may be compared with previous transient absorption measurements reported in aqueous solution and in acetonitrile at similar excitation wavelengths. Interestingly, for the thiobases with emission and transient absorption data at similar excitation wavelengths, collected in Table 1 and Table 2, the fluorescence lifetimes seem to be comparable to the corresponding intersystem crossing lifetimes measured using transient absorption spectroscopy. For instance, 2tUra and 2,4dtUra in PBS exhibit fluorescence lifetimes of 0.18 and 0.12 ps, respectively, exciting at 267 nm, which are in agreement with the 0.167 and 0.136/0.217 ps reported recently for the decay of the ^1^π_S_π* bright states of 2tUra and 2,4dtUra, respectively, upon 270 nm excitation [28]. However, they are smaller than the 0.35 and 0.22 ps intersystem crossing lifetimes exciting at 316 and 350 nm [29,30], respectively (Table 1). 

The most obvious reason for the differences observed in the ultrafast fluorescence and transient absorption lifetimes is that different excitation wavelengths and time resolutions were used in those works. However, it should also be kept in mind that transient absorption and time-resolved fluorescence are complementary techniques that often provide time-resolved information from different decay processes [41,42]. While fluorescence up-conversion is sensitive to states with significant radiative transition probability, transient absorption can, in principle, monitor all excited-state populations, but particularly those that are not emissive (i.e., states with small radiative transition cross sections). In fluorescence up-conversion, the excited state population (wavepacket) can only be observed for as long as the overlap with the ground state is preserved.

As recently reviewed by Ashwood et al. [26], most of the transient absorption measurements reported for the thiobase derivatives have primarily focused on measuring the ultrafast intersystem crossing dynamics to the triplet manifold upon UVA excitation. Assisted by high-level computational methods, reviewed recently by Corral and co-workers [27], these investigations have put forward the idea that direct excitation of the lowest-energy ^1^π_S_π* spectroscopic state in the thiobases primarily result in ultrafast internal conversion to the lowest-energy ^1^n_S_π* state, from which a large fraction of the population intersystem crosses to the triplet manifold in a matter of hundreds of femtoseconds. Other intersystem crossing pathways, directly from excited ^1^π_S_π* states to upper lying triplet states, have also been predicted by static and dynamic simulations [28,35,39,43,44]. Collectively, the experimental and computational results support the idea that non-emissive states primarily act as doorway states in the ultrafast intersystem crossing pathway from the corresponding ^1^π_S_π* states to the low-lying ^3^π_S_π* states. This premise is further supported by recent sub-20 fs transient absorption and computational measurements performed by Cerullo, Garavelli, and co-workers for 2tUra, 4tUra, and 2,4dtUra in aqueous solution [28,38]. 

In the following sections, we discuss how the excitation wavelength, position, and degree of thionation, as well as the formation of the N-glycosidic bond and solvent, affect the fluorescence lifetimes of selected thiopurine and thiopyrimidine derivatives. Finally, we briefly compare the ultrafast fluorescence lifetimes reported for the canonical nucleobases to those presented for the thiobase derivatives in this study. General conclusions are provided in Section 5. 

### 3.1. Effect of the Excitation Wavelength on the Fluorescence Lifetimes 

Recent investigations in the gas phase [40] and in solution [28,39,40] have demonstrated that the excited-state dynamics of the thiobases is modulated by the excitation wavelength used. Hence, we have investigated the effect of the excitation wavelength on the fluorescence decay lifetime of a selected subgroup of thiobases in both PBS and acetonitrile solutions (Table 1 and Table 2). Interestingly, the ultrafast fluorescence lifetimes reported in PBS in Table 1 barely change upon excitation at 267 or at 362 nm within the experimental uncertainties. They also seem to be comparable to the ultrafast transient absorption lifetimes reported in the literature for 2tUra, 4tThd, and 2,4dtUra exciting at 270 nm [28,39]. In acetonitrile, however, the fluorescence lifetime seems to increase, going from 267 to 362 nm excitation. We caution, however, that the emission was detected at 380 nm when excitation was performed at 267 nm, while it was detected at 425 nm when excitation was done at 362 nm. Hence, we cannot rule out that vibrational and/or solvation dynamics may have contributed to the reported fluorescence lifetimes (see also Table S1). It is evident that additional, systematic fluorescence up-conversion investigations, ideally reporting time-resolved spectra, are necessary before attempting to arrive at any major conclusions from the data collected in Table 1 and Table 2. This also seems to be the case for the transient absorption data reported in these tables. Regardless of these limitations, we consider that the fluorescence lifetimes reported in this study provide valuable, new information about the excited-state dynamics of these relevant biomolecules, which we envision will stimulate further experimentation.

### 3.2. Effects of N9-Glycosylation on the Fluorescence Lifetime of 6tGua 

The excited-state dynamics of 6tGua and 6tGuo have been investigated in reasonable detail, using transient absorption spectroscopy [25,31,34,37] and theoretical calculations [27,43,45,46]. It has been shown that excitation to the lowest-energy ^1^π_S_π* state results in a bifurcation process in which a small fraction of the population internally converts to the ground state, while most of the population intersystem crosses to the triplet state in an ultrafast time scale [34,37]. The internal conversion to the ground state is thought to occur through a ^1^π_S_π* → ^1^n_S_π* → S_0_ pathway, while the intersystem crossing to the triplet manifold has been proposed to proceed through two parallel relaxation pathways involving ^1^π_S_π* and ^1^n_S_π* minima [43,45].

The results shown in Figure 1 indicate that the N9-glycosylation of 6tGua leads to a 28% decrease in the fluorescence lifetime upon excitation at 267 nm. Using broadband transient absorption spectroscopy, Ashwood et al. [37] recently showed that the intersystem crossing lifetime of 6tGua decreased by 45%, compared to that of 6tGuo when excitation is performed at 345 nm [34]. Collectively, the fluorescence up-conversion and transient absorption results suggest that the N9-glycosylation of 6tGua leads to both a faster decay of the ^1^π_S_π* population and a faster population of the triplet state in 6tGuo (τ_ISC_ = 0.56 ± 0.06 ps for 6tGua [37] versus τ_ISC_ = 0.31 ± 0.05 ps for 6tGuo [34]). The fluorescence lifetimes exciting at 267 nm suggest that the rate of fluorescence decay of 6tGua increases upon N-glycosylation in PBS.

### 3.3. Fluorescence Decay Lifetimes of the Thiopyrimidine Monomers 

Recent steady-state results, transient absorption, and computational investigations have shown that the photophysical and dynamical properties of the thiopyrimidine derivatives are modulated by both the position and degree of thionation, as well as by the addition of ribose or deoxyribose at the N1 position [25,26,27,28]. Thionation of the pyrimidine bases stabilizes the lowest-energy ^1^π_S_π* and ^1^n_S_π* excited states, with the ^1^n_S_π* state always being the lowest-lying singlet state, while simultaneously increasing the intersystem crossing efficiency to the triplet manifold. The decay signals depicted in Figure 2 show that the fluorescence lifetimes of the thiopyrimidine derivatives in aqueous solution also depends on both the position and degree of thionation, as well as on the addition of a N1-glycosidic group (Table 1). For example, the ultrafast fluorescence decay signal of 2tCyt seems to decay in a faster time scale than that of 2tCyd, but for 2tUra and 2tUrd, the lifetimes are within experimental uncertainties. Interestingly, both 2tUrd and 2tCyd exhibit a second, small-amplitude, longer-lived fluorescence lifetime of a few picoseconds (Table 1), which is not detected for either 2tUra or 2tCyt. 4tThd also exhibits a second, small amplitude picosecond lifetime, but we do not have the corresponding data for 4tThy. As discussed in the next paragraph, the bi-exponential fluorescence decay may be due to the presence of two minima in the ^1^π_S_π* potential energy surface [39] or to the simultaneous population of two ^1^π_S_π* states that decay in parallel upon excitation at 267 nm [28]. 

In the case of 4tUra, however, sugar addition at the N1 position has barely any effect on the fluorescence lifetime (Figure 2b and Table 1). Figure 2b also shows that the fluorescence decay dynamics of 4tUrd is quite different from that of 4tThd. While both 4tUrd and 4tThd exhibit almost identical sub-picosecond fluorescence lifetimes (Table 1), only 4tThd exhibits a second, few-picosecond fluorescence lifetime similar to 2tUrd and 2tCyd. Recent QM/MM calculations, coupled with multireference CASPT2//CASSCF, suggest that 2tUra simultaneously populates two ^1^π_S_π* states upon excitation at 270 nm, which decay in parallel in order to populate the triplet manifold [28]. One of the ^1^π_S_π* minima is predicted to be planar, while the other exhibited a twisted conformation with the sulfur atom out of the molecular plane [28]. The presence of a small-amplitude, secondary fluorescence lifetime in these three thiopyrimidine nucleosides could also be explained by the presence of more than one minimum in one or more of the excited ^1^π_S_π* potential energy surfaces. Static and dynamic quantum-chemical calculations, performed by Martínez–Fernández et al. [39] for 4tThy in water, predicts the population of planar and twisted minima in the lowest-energy ^1^π_S_π* potential energy surface. It is conceivable that the bi-exponential fluorescence decay in 2tUrd and 4tThd may be associated with the simultaneous population of two ^1^π_S_π* states upon excitation at 267 nm or with the population of two minima in a ^1^π_S_π* potential energy surface in PBS. The observation that 4tThd exhibits a small-amplitude, picosecond lifetime upon excitation at 362 nm (Table 1), in addition to the primary sub-1 ps fluorescence lifetime, lend some support to the mechanism proposed by Martínez–Fernández et al. [39]. However, one must wonder why 2tUra, or any other thiobase in Table 2, does not exhibit bi-exponential fluorescence lifetimes. We also remark that the picosecond fluorescence lifetimes reported in Table 1 for 2tCyd, 2tUrd, and 4tThd only contribute to no more that ca. 26% of the total fluorescence signal and have large errors due to the limited time window used. Furthermore, the calculations by Teles–Ferreira et al. [28] and Martínez–Fernández et al. [39] were performed for the nucleobases (2tUra and 4tThy, respectively), not the nucleosides, and our experimental results reported in Table 1 suggest that the sugar moiety could play a role.

The fluorescence lifetimes of 0.28 ± 0.03 ps and 0.24 ± 0.08 ps reported for 4tThd in Table 1, upon excitation at 267 and 362 nm, respectively, seem to correlate with the intersystem crossing lifetime of 0.21 ± 0.04, 0.25 ± 0.05, and 0.24 ± 0.02 ps, reported using transient absorption in aqueous solution and exciting at 270, 334, and 340 nm, respectively [33,39]. It is also in good agreement with the fluorescence decay signal previously reported for 4tThd in aqueous buffer solution upon excitation at 266 nm [25]. In an early report, it was shown that more than 90% of the fluorescent population decayed in 0.23 ± 0.03 ps. A second lifetime of a few picoseconds was also extracted, which accounted for less than 10% of the total fluorescence intensity. In addition, a zero time anisotropy of ≤0.2 was measured [25]. This relatively small value suggests that the emitting transition dipole is not parallel to the absorbing one, indicating that an electronic relaxation occurs within the time resolution of the experimental setup. Transient absorption investigations for 4tThd and for 2tUra, 4tUra, and 2,4dtUra, lend support to the idea that a portion of the initial excited-state population relaxes in sub-200 fs [10,28,30,32,33,38,39,47], which is independent of the excitation wavelength used.

Figure 2c demonstrates that both the position and degree of thionation affect the fluorescence lifetimes of the thiouracil derivatives in PBS. Importantly, the fluorescence lifetimes in the thiouracil series decreases as 4tUra > 2tUra > 2,4dtUra (Table 1), which is a similar trend to that observed for the intersystem crossing lifetimes of both the thiouracil [28,30] and the thiothymine [10] series. Figure 2a,d shows that 2tCyt, 2tUra, 2tUrd, 2,4dtThy, 2,4dtUra, and 2,4dtUrd exhibit the fastest fluorescence lifetimes of the thiobases investigated in this study in aqueous solution. Similar results are observed in acetonitrile (Table 2).

### 3.4. Solvent Effects on the Fluorescence Decay Lifetimes

The fluorescence up-conversion measurements provide the first determinations of the excited-state lifetimes of seven thiobases in acetonitrile, which are collected in Table 2. In particular, the fluorescence signals depicted in Figure 4 evidence the fact that the magnitude of the fluorescence lifetimes for 4tThd, 4tUrd, and 4tUra increases, going from a protic to an aprotic polar solvent. Specifically, a 16%, 46%, and 55% slowdown in the fluorescence lifetimes of 4tThd, 4tUrd, and 4tUra, respectively, are estimated, going from PBS to acetonitrile. This result suggests that hydrogen bonding, and/or an increase in the dielectric constant of the solvent, affects the energy and the topography of the excited-state potential energy surfaces of these thiopyrimidine monomers, in such a way that nonradiative decay in PBS is accelerated [25,27,40,48,49]. These results are consistent with femtosecond transient absorption experiments that have demonstrated an increase in the intersystem crossing rate to populate the triplet manifold for 6tGuo [34], 2tThy [29], 4tThd [10,32,33], and 2tUra [29,30] in going from acetonitrile to PBS [25,27]. 

The effect of the position of thionation on the fluorescence lifetime of thiouracil is very different in PBS than in acetonitrile. While there is hardly any difference between 2tUra and 4tUra in PBS (Figure 2b), the fluorescence decay of the latter is significantly slower in acetonitrile (Figure 3a). In addition, the fluorescence up-conversion results reported in Table 2 demonstrated that the substitution of the hydrogen atom at the N1 position of 4tUra by a sugar moiety increases the fluorescence lifetime by 26% in acetonitrile (Figure 3c). This is contrary to what is observed in PBS (Figure 2c), where no effect is observed. Similarly, the effect of 5-methylation of 4tUrd (i.e., 4tThd) is very different in PBS compared to that in acetonitrile. In the former case, the fluorescence decay of 4tThd is much slower than that of 4tUrd (Figure 2c). In acetonitrile, however (Figure 3c), the fluorescence decay of 4tThd is slightly faster than that of 4tUrd, and is nearly as fast as that of 4tUra. These results show a subtle influence of the solvent on the excited state dynamics. Indeed, the solvent can play an important role in modifying the depth of the potential energy minima and the magnitude of the energy barriers that restrict access to key conical intersections and relevant deactivation pathways [50,51,52]. For instance, such effects have been observed for the canonical nucleobases even though the topography of the excited-state potential energy surfaces is significantly different [53,54,55]. The solvent can also affect the vibronic coupling between singlet and triplet excited states by altering their energy gaps [50,56], which can ultimately modulate the decay lifetimes and the yields of competing emissive and intersystem crossing decay pathways, possibly overriding the decelerating effect of the N-glycosylation. Theoretical calculations are needed to better understand how the solvent affects the topography of the excited-state potential energy surfaces and the dynamics of the thiobases.

It should be remarked that longer-lived (i.e., a few picoseconds) fluorescence lifetimes are not observed for any thiobases in acetonitrile, in contrast to what is the case for 2tCyd, 2tUrd, and 4tThd in PBS. As discussed in Section 3.3, this may be an indication of an additional ^1^π_S_π* minimum in these thiobases in PBS, which communicate less effectively with or has a higher energy barrier for accessing the triplet manifold. Additional theoretical calculations in acetonitrile are needed in order to fully understand the origin of this phenomenon. 

Concerning the double-substituted thiobases, the fluorescence signals decay faster than those of the mono-substituted ones (Figure 2 and Figure 3), both in PBS (Table 1) and in acetonitrile (Table 2 and Table S1). Similarly, the N-glycosylation of 2,4dtUra has no noticeable effect on the fluorescence decay signal (Figure 2d and Figure 3c). These observations suggest that dithionation saturates the density of singlet and triplet states and minimizes the energy gaps in such a way that nonadiabatic decays among singlets and triplet states are maximized in the dithionated nucleobases, and hence, N-glycosylation cannot further increase the nonadiabatic rates. Here, again, quantum-chemical and dynamics simulations can significantly aid in developing a rigorous understanding of these phenomena.

Finally, it should be noted that the effect of N-glycosylation on the fluorescence lifetime of 2tCyt and 4tUra in PBS and acetonitrile is the opposite to that of 6tGua and 2tUra within experimental uncertainties (Table 1 and Table 2). Recent transient absorption investigations for 2tCyt [26], 2tThy [10,57], and 2tUra [26] in PBS demonstrated that the rates of nonradiative decay increase when a sugar substitute is added to the thiobase to form the nucleoside derivatives. However, the effect of N-glycosylation on the transient absorption decay signals of 4tUra, or on the fluorescence lifetimes of 6tGua in acetonitrile, have yet to be reported. Additional transient absorption and fluorescence up-conversion investigations are required, preferably with higher time resolution, to reach any conclusions. 

### 3.5. Comparison with DNA and RNA Canonical Nucleobases

The fluorescence up-conversion results show that thionated nucleobases exhibit fluorescence lifetimes of a few hundreds of femtoseconds upon excitation at 267 and at 362 nm (Table 1), which are remarkably similar to the fluorescence lifetimes reported for the canonical nucleobases [58]. However, photoexcited nucleobases primarily decay by ultrafast internal conversion to the ground state [59,60], whereas photoexcited thiobases predominantly populate long-lived and reactive triplet states with near unity yields [25,26]. Sulfur-substitution significantly lowers the energies of the ^1^nπ* and ^1^ππ* minima by up to 1 eV, compared to the canonical nucleobases [35], while the energy of the lowest conical intersections (CI) stays approximately constant upon thionation. Calculations predict that the reason behind this key differentiation is that thionation of the nucleobases stabilizes all the singlet and triplet nπ* and ππ* states in which the excitation is primarily localized on the sulfur atom, whereas other states, and their corresponding CIs, are not stabilized to a similar extent [27,35]. Indeed, the population in the excited ^1^π_S_π* states of the thiobases rapidly decay to ^1^n_S_π* or ^3^n_S_π* minima, which are almost isoenergetic to one or more states and exhibit large spin-orbit couplings [27,28,35]. This, in turn, enables efficient fluorescence quenching through ultrafast intersystem crossing to reactive triplet states [25,26,35]. Therefore, while both canonical and thionated nucleobases exhibit ultrafast fluorescence quenching, the canonical nucleobases are unusually photostable (with minor yields of triplet state population only observed for the pyrimidine monomers in solution) [61,62,63,64,65,66], while the thionated nucleobases are highly photochemically reactive. We find it remarkable that a single atom substitution can so effectively switch on or off the photoreactivity of the building block of life [26]. 

## 4. Materials and Methods 

### 4.1. Chemicals

Phosphate buffered saline (PBS) solution with a pH of 7.4 with a total salt concentration of 50 mM was made using monosodium and disodium phosphate salts (99.0% purity, Sigma–Aldrich, St. Louis, MO, USA). The pH was adjusted with 0.1 M sodium hydroxide and phosphoric acid (Fisher Chemical, Fisher Scientific, Leicestershire, UK, certified ACS grade ≥85% *w*/*w*) to the desired pH (±0.1 pH units). 4-thiothymidine (99% purity), 2-thiouridine (95–98% purity) were purchased from Carbosyn Limited, Berkshire, UK. 6-Thioguanine (≥98% purity), 6-thioguanosine (97% purity), 2-thiocytosine (97% purity), 4-thiouracil (97% purity), 4-thiouridine (≥99% purity), 2-thiouracil (≥99% purity), 2,4-dithiouracil (98% purity), 2,4-dithiothymine (purity not reported, but no fluorescent impurity was detected in this work), and acetonitrile (HPLC grade, ≥99.93%) were purchased from Sigma–Aldrich. 2-Thiocytidine (≥97% purity) was purchased from Berry & Associates, Inc. (Dexter, MI, USA) and 2,4-dithiouridine (95–98% purity) was purchased from Biosynth–Carbosynth (Compton, Berkshire, UK). All thiobase monomers were used as received. 

### 4.2. Steady-State Absorption and Emission

Room temperature absorption spectra were recorded using a Perkin Elmer Lambda 900 UV-vis-NIR spectrometer (PerkinElmer Inc., Hopkinton, MA, USA). The thiobases absorption for a path length of 1 mm at 267 or at 362 nm ranged from 0.2 to 0.5, and from 0.3 to 0.8, depending on the solubility and solvent used. Samples were prepared in approximately 30 mL batches for use in a flow cell with an optical path length of 1 mm.

Emission spectra of the thiobases in PBS were measured in a Cary Eclipse spectrofluorimeter (Varian Inc., Palo Alto, CA, USA) at room temperature under air, O_2_, or N_2_-saturated conditions, and were corrected for the spectral sensitivity of the spectrofluorimeter and the solvent background signal (Figures S2 and S3). For 4tUrd, the emission spectrum was also collected in acetonitrile. The absorbance of the thiobases was in the range of 0.18 and 0.20 in a 1 cm optical path cell at the excitation wavelength of 268 nm. The emission spectra were collected using a scan rate of 30 nm/min, a data interval of 0.5 nm, and an averaging time of 1 s. The excitation and emission slit widths were set to 5 nm and the photomultiplier tube (PMT) gain to −800 V. 

### 4.3. Femtosecond Fluorescence Spectroscopy

The fluorescence up conversion setup has been described in detail elsewhere [53,67]. For the excitation, either the second (362 nm) or third (267 nm) harmonic of the Ti:sapphire laser (120 fs FWHM) (Coherent Inc., Santa Clara, CA, USA) was used. In the former case, the fundamental Ti:sapphire laser frequency was tuned to 724 nm with an average power of 1.4 W in the mode-locked regime. The 362 nm were generated in a 0.5 mm Type I BBO crystal. In the latter case, the fundamental Ti:sapphire laser frequency was tuned to 800 nm with an average power of 2 W in the mode-locked regime. The 267 nm excitation pulses were produced by first generating the second harmonic in the first crystal and was then mixed with the residual 800 nm fundamental beam in the second crystal. In both cases, 0.5 mm Type I BBO crystals were used, resulting in a tripling efficiency of about 5%. For both 362 and 267 nm excitations, the average excitation powers of 5 to 20 mW were used, depending on the sample, corresponding to less than 0.1 GW/cm^2^ [54]. The fluorescence decays at different wavelengths were made for parallel (*I_par_*) and perpendicular (*I_perp_*) excitation/emission configurations and the isotropic decay was calculated using the equation *F(t) = I_par_(t) + 2I_perp_(t)*.

The excitation pulse (267 or 362 nm) was passed through an appropriate half-wave plate before entering the sample cell in order to control the polarization relative to that of the gating pulse. The fluorescence from the sample cell was collected and focused on the sum-frequency crystal using parabolic off-axis mirrors. For both 267 and 362 nm excitations, the residual 800 nm fundamental was used as the gate pulse and passed through a mechanical delay line before being focused on the sum-frequency crystal. 

The sum-frequency generation was achieved by spatially overlapping the fluorescence emission of the sample with the gate pulse (800 nm) in the up-conversion BBO crystal. The phase matching angle was optimized for the chosen fluorescence wavelength, in this case 380 or 425 nm. The resulting sum frequency signal was spectrally filtered with a double monochromator and was detected by a PMT (Hamamatsu 1527P, −1250 V) connected to a photon-counter (Stanford SR 400). The time-evolution was obtained by controlling the relative delay of the gate pulse with regards to the excitation pulse via the mechanical delay line. Typically, a time window of a few picoseconds was scanned with a step of a few tens of femtoseconds. In order to improve the signal quality, several scans were averaged before data processing. The instrument response function (IRF) was estimated to 300 fs (FWHM) from the Raman signal of water and was deconvoluted from the fluorescence decay signals using a Gaussian-shaped model (see below).

### 4.4. Data Analysis

Data analysis of the fluorescence up-conversion was performed using Igor Pro 6.32A software (Wavemetrics, Inc., Lake Oswego, OR, USA) and a homemade Fortran program. The lifetimes obtained were from the isotropic decay measurements at a wavelength of 380, 425, 450, 525, or 532 nm, exciting the thiobases at 267 or 362 nm, respectively, in both PBS and acetonitrile. The florescence decay lifetimes for the thiobase monomers were fitted with a Gaussian instrument response function with a full width at a half maximum (FWHM) of 300 fs and a single or double exponential function when appropriate. Depending on the signal-to-noise ratio, reliable lifetimes as short as one tenth of the IRF can be determined from such a fitting [68].

We are currently more modest in regards to the time-resolution of our setup because the up-conversion signals, were, in most cases, low. We judged it to be about 100 fs after the nonlinear fitting/deconvolution procedure. However, we noted that, given the temporal resolution of our setup, as well as the very short lifetimes for most of the thiobases, the fluorescence lifetimes might have been slightly biased because of the precise fitting algorithm used (i.e., analytical or numerical), how the IRF was described (i.e., analytical, Gaussian, experimental, Raman, etc.), and by the choice of the fluorescence decay model function (i.e., mono-exponential, multi-exponential, rate equations, etc.). For these reasons, the lifetimes reported in Table 1, Table 2, and Table S1 should be viewed as a comparable set of data rather than absolute values.

## 5. Conclusions

In this contribution, we report the first comprehensive set of fluorescence lifetimes for twelve thiopurine and thiopyrimidine derivatives in both PBS and acetonitrile, as well as exciting at two different wavelengths. All the primary fluorescence decays are ultrafast and are characterized by sub-picosecond lifetimes. It is shown that the position and degree of thionation, the formation of the N-glycosidic bond, and the solvent environment affect the magnitude of their fluorescence lifetimes. Several of the thiobases exhibit slower fluorescence decay when excitation is performed at 362 versus 267 nm in acetonitrile, while the fluorescence lifetimes are practically excitation-wavelength independent (i.e., 267 versus 362 nm) for the majority of the thiobases investigated in both solvents. Thiobases exhibit both femtosecond fluorescence quenching and femtosecond intersystem crossing lifetimes. Hence, efficient internal conversion from initially populated ^1^π_S_π* states to ^1^n_S_π* or ^3^n_S_π* minima seems to primarily deactivate the spectroscopic singlet states in these molecules and lead to the population of long-lived triplet states. As the calculations show that ^1^n_S_π* and ^3^n_S_π* minima are close in energy to one or more excited states that are strongly coupled by large spin-orbit factors [27,28,35], subsequent intersystem crossing to long-lived and reactive triplet states is facilitated [26]. Collectively, the fluorescence up-conversion results reported in this study lend support to the electronic relaxation mechanisms proposed in the literature for the thiobase derivatives [26,27], and elucidate the vanishingly small fluorescence yields reported for the thiobases [25]. 

## Figures and Tables

**Table 1:** Fluorescence (Fl) and transient absorption (TA) lifetimes of the thiopyrimidine and thiopurine derivatives in PBS at pH 7.4. The relative amplitudes for biexponential lifetimes are reported within parentheses. Uncertainties for the fluorescence lifetimes correspond to one standard deviation.

Thiobase	Fl Lifetime (ps) ^a^ λ_exc_ = 267 nm	TA Lifetime (ps) ^b^ λ_exc_ = 270 nm	Fl Lifetime (ps) ^c^ λ_exc_ = 362 nm	TA Lifetime (ps) ^d^ λ_exc_ = UVB/UVA
2tCyt	0.11 ± 0.02			0.21 ± 0.05 [35]
2tCyd	0.19 ± 0.07 (0.74) ^e^ 1.9 ± 0.7 (0.26) ^e^			0.20 ± 0.05 [36]
6tGua	0.27 ± 0.02			0.56 ± 0.06 [37]
6tGuo	0.19 ± 0.01			0.31 ± 0.05 [34]
2tUra	0.18 ± 0.02	0.167 [28]		0.35 ± 0.06 [29]
2tUrd	0.14 ± 0.02 (0.94) 4 ± 3 (0.06) ^e^			0.25 ± 0.04 [36]
4tUra	0.24 ± 0.02			0.24 ± 0.02 [30] 0.076 ± 0.016 [38]
4tUrd	0.26 ± 0.02			
4tThd	0.28 ± 0.03 (0.94) 12 ± 7 (0.06) ^e^	0.21 ± 0.04 [39]	0.24 ± 0.08 (0.98) 2.6 ± 0.5 (0.02) ^e^	0.24 ± 0.02 [33] 0.25 ± 0.05 [39]
2,4dtThy	0.16 ± 0.02		0.16 ± 0.02	0.18 ± 0.04 [10]
2,4dtUra	0.12 ± 0.02	0.136 [28], 0.217 [28]	0.11 ± 0.01	0.22 ± 0.04 [30]
2,4dtUrd	0.14 ± 0.03		0.09 ± 0.01	

^a^ fluorescence detected at 380 nm upon excitation at 267 nm; ^b^ only the sub-1 ps lifetimes reported from the transient absorption results upon excitation of the thiobases at 270 nm are shown; ^c^ fluorescence detected at 425 nm upon excitation at 362 nm; ^d^ only the sub-1 ps lifetimes reported from the transient absorption results upon excitation of the thiobases in the UVB/UVA region are shown (see original work for experimental details); ^e^ this fluorescence lifetime should be taken with caution due to the small amplitude of the component and/or the limited time-window used.

**Table 2:** Fluorescence (Fl) and transient absorption (TA) lifetimes of the thiopyrimidine derivatives in acetonitrile. Uncertainties for the fluorescence lifetimes correspond to one standard deviation.

Thiobase	Fl Lifetime (ps) ^a^ λ_exc_ = 267 nm	TA Lifetime (ps) ^b^ λ_exc_ = 268/270 nm	Fl Lifetime (ps) ^c^ λ_exc_ = 362 nm	TA Lifetime (ps) ^d^ λ_exc_ = 316/340 nm
2tUra	0.14 ± 0.03	0.44 ± 0.03 [40]		0.34 ± 0.09 [29]
4tUra	0.41 ± 0.03		0.84 ± 0.03	
4tUrd	0.66 ± 0.03		0.72 ± 0.02	
4tThd	0.45 ± 0.02	0.19 ± 0.04 [39] 0.49 ± 0.09 [39]	0.50 ± 0.03	0.54 ± 0.01 [32]
2,4dtThy	0.11 ± 0.02		0.15 ± 0.03	
2,4dtUra	0.16 ± 0.02		0.23 ± 0.03	
2,4dtUrd	0.17 ± 0.02		0.18 ± 0.03	

^a^ fluorescence detected at 380 nm upon excitation at 267 nm; ^b^ only the sub-1 ps lifetimes reported from the transient absorption results upon excitation of thiobases at 268 or 270 nm are shown; ^c^ fluorescence detected at 425 nm upon excitation at 362 nm (see Table S1 for fluorescence lifetimes at other emission wavelengths); ^d^ only the sub-1 ps lifetimes reported from the transient absorption results upon excitation of thiobases in the UVB/UVA region are shown (see original work for experimental details).

## Supplementary Materials

The following are available online. Absorption and emission spectra of the investigated thiobase monomers in PBS at pH 7.4 or in acetonitrile. Table with fluorescence lifetimes for selected thiobases in acetonitrile exciting at 362 nm and detecting at 450, 525 or 532 nm.
