# Supplementary material for: Excited State Lifetimes of Sulfur-Substituted DNA and RNA Monomers Probed Using the Femtosecond Fluorescence Up-Conversion Technique
Source: Molecules. 2020 Jan 29;25(3):584. doi: 10.3390/molecules25030584 (PMC7037914; doi:10.3390/molecules25030584)
Supplement: Supplementary file 1 [file molecules-25-00584-s001.pdf]

Excited State Lifetimes of Sulfur-Substituted DNA and RNA Monomers Probed Using the Femtosecond Fluorescence Up-Conversion Technique

Matthew M. Brister[1,†] (ORCID: 0000-0002-1563-6496), Thomas Gustavsson[2,*] (ORCID: 0000-0001-7030-6812), Carlos E. Crespo-Hernández[1,*] (ORCID: 0000-0002-3594-0890)

[1] Department of Chemistry, Case Western Reserve University, 10900 Euclid Ave., Cleveland, Ohio 44106, United States.

[2] LIDYL, CEA, CNRS, Université Paris-Saclay, F-91191 Gif-sur-Yvette, France.

[†] Present address: Lawrence Berkeley National Laboratory, Atomic Molecular and Optical Sciences Experimental Group, Chemical Sciences Division, Berkeley, California 94720, United States.

* Authors to which correspondence should be addressed; thomas.gustavsson@cea.fr, carlos.crespo@case.edu

Supplementary Material

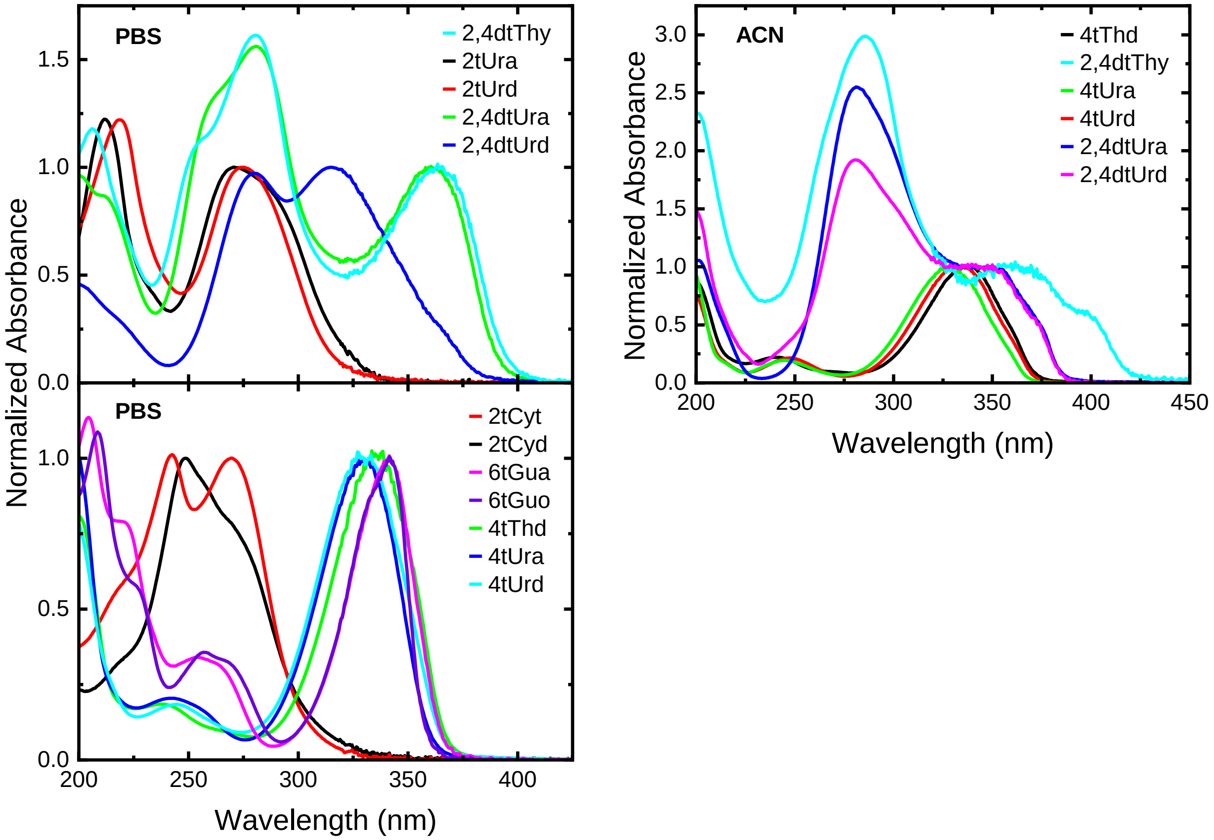

Figure S1. Normalized ground-state absorption spectra of the investigated thiobase monomers in phosphate buffer saline (PBS) solution at pH 7.4 and in acetonitrile (ACN).

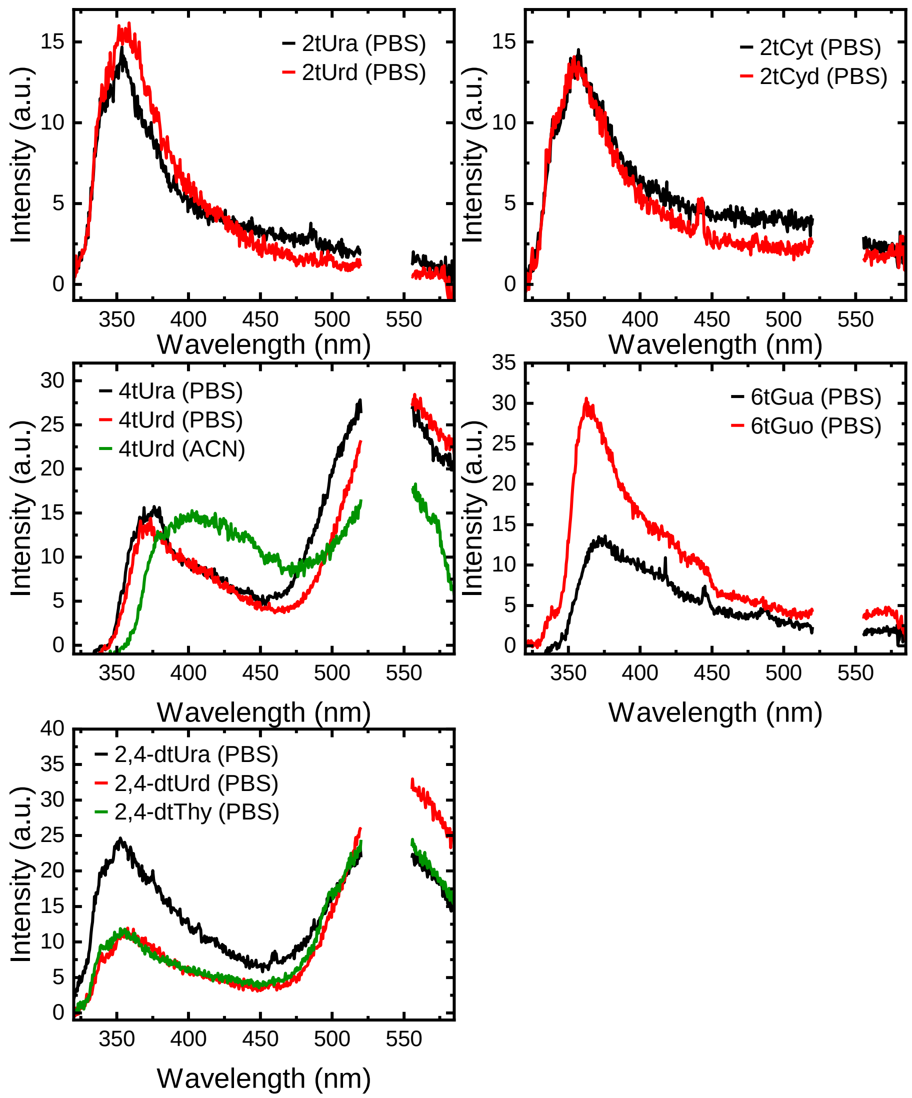

Figure S2. Emission spectra of the investigated thiobase monomers in phosphate buffer saline (PBS) solution at pH 7.4 or in acetonitrile (ACN). Excitation was performed at 268 nm in air-saturated conditions. The overtone at ca. 536 ± 15 nm has been masked in each plot. Other experimental and instrument conditions are reported in Section 4.2. The additional emission bands observed for 4tUra, 4tUrd, 2,4dtUra, 2,4dtThy around 550 nm are assigned to phosphorescence emission because their intensity increases/decreases when the solutions are purged with N_2/O_2 (see also Figure S3 below). Dual emission (i.e., fluorescence + phosphorescence) has been reported previously for 4tThd in different solvents.[1-2]

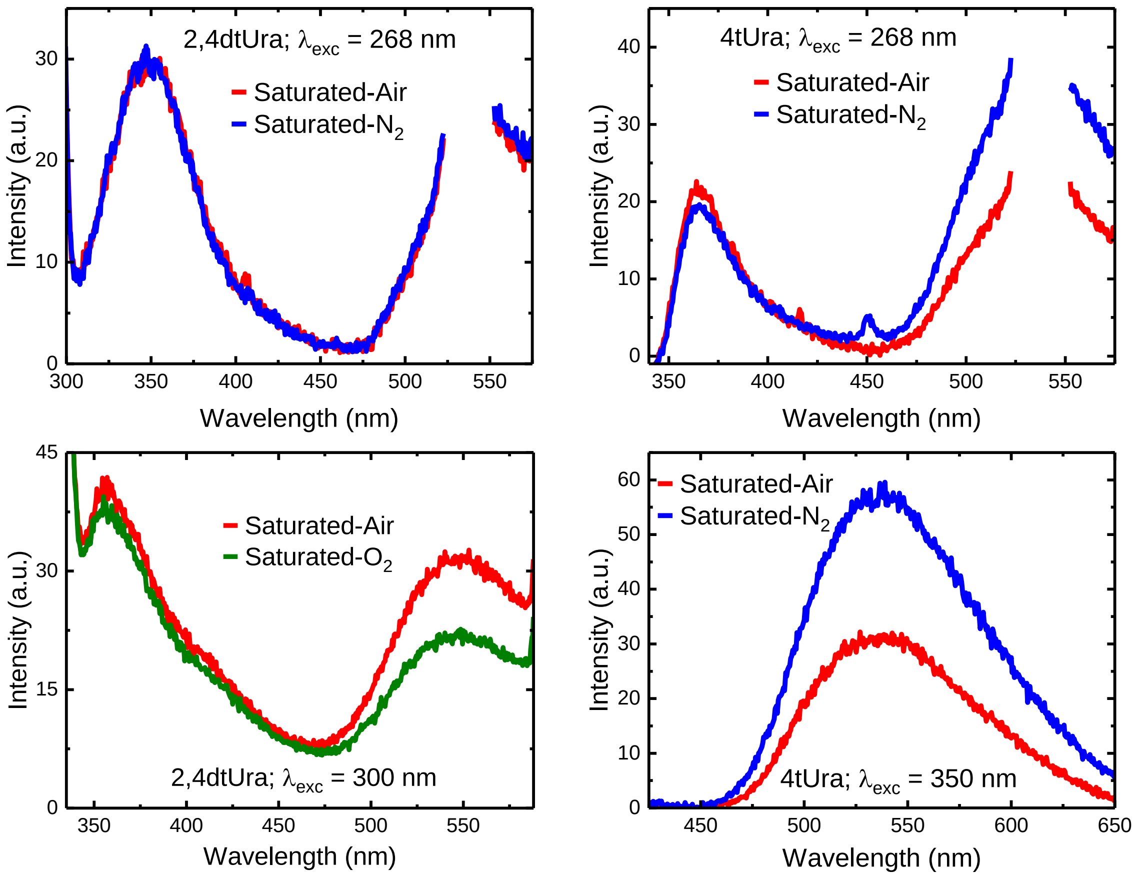

Figure S3. Emission spectra for 2,4dtUra and 4tUra in phosphate buffer saline (PBS) solution at pH 7.4 at selected excitation wavelengths and under N_2-, air-, or O_2-saturated conditions. The overtone at ca. 536 ± 15 nm has been masked from the top panels. We note that a recent study has assigned the emission band at ca. 550 nm in 2,4dtUra to fluorescence from a high-energy $^1\pi_S\pi^*$ state,[3] in contrast to our assignment to phosphorescence emission from the $^3\pi_S\pi^*$ state for both 4tUra and 2,4dtUra.

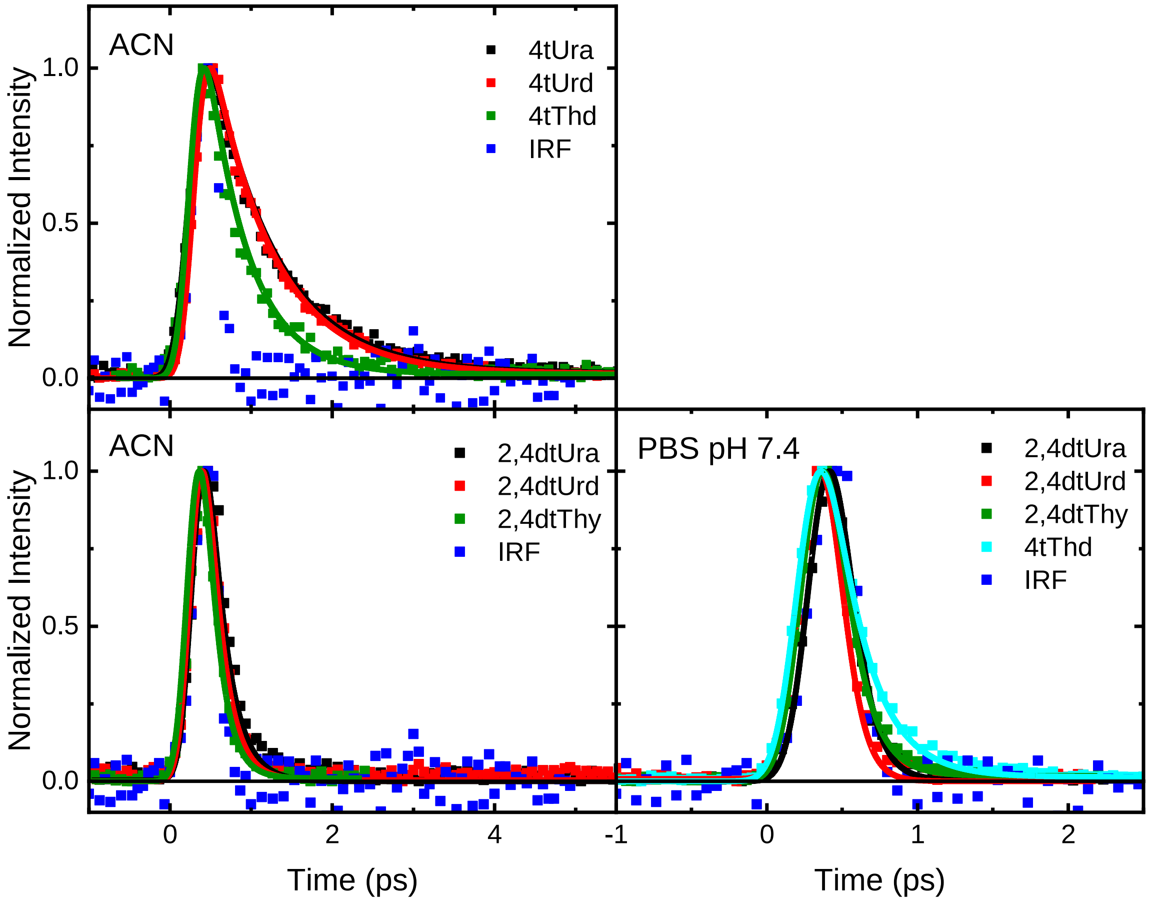

Figure S4. Normalized isotropic fluorescence up conversion signals for selected thiobases in acetonitrile (ACN) or in phosphate buffer saline (PBS) solution at pH 7.4 at the emission wavelength of 425 nm upon excitation at 362 nm.

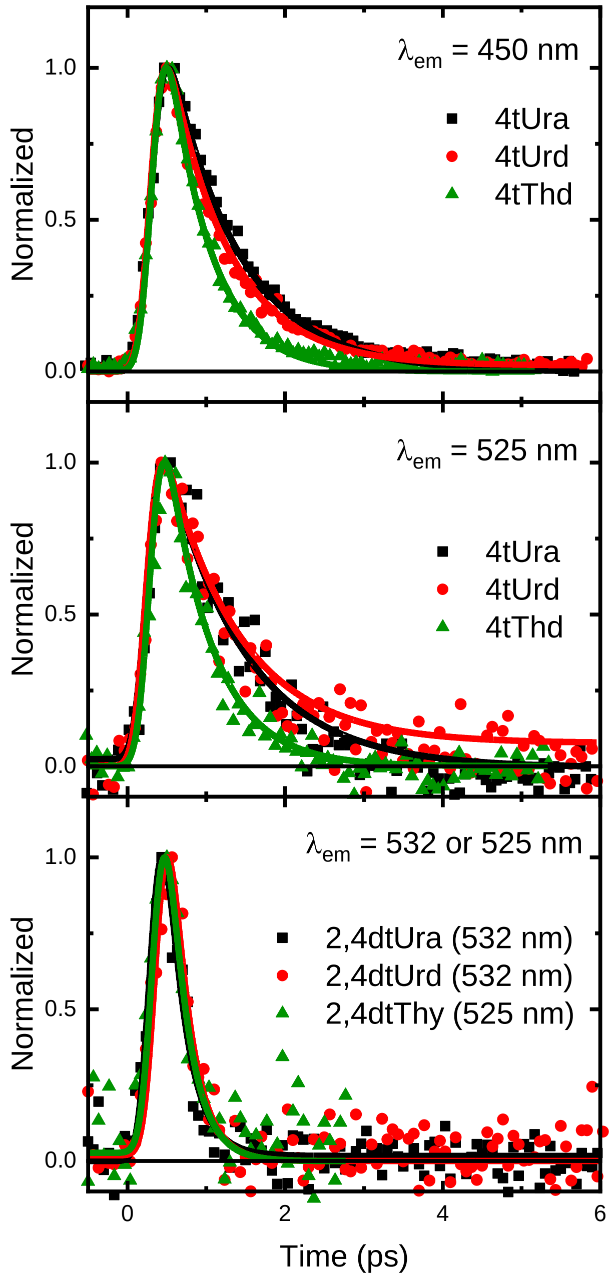

Figure S5. Normalized isotropic fluorescence up conversion signals for selected thiobases in acetonitrile at the emission wavelengths of 450, 525, or 532 nm upon excitation at 362 nm.

Table S1. Fluorescence lifetimes of selected thiobases in acetonitrile.

λ_{exc} = 362 nm IRF: 300 fs	λ_{em} = 425 nm Isotropic (ps)	λ_{em} = 450 nm Isotropic (ps)	λ_{em} = 525 nm Isotropic (ps)	λ_{em} = 532 nm Isotropic (ps)
4tUra	0.84 ± 0.03	0.89 ± 0.04	0.9 ± 0.2	-
4tUrd	0.76 ± 0.02	0.76 ± 0.02	0.93 ± 0.10	-
4tThd	0.50 ± 0.03	0.51± 0.02	0.55 ± 0.05	-
2,4dtThy	0.16 ± 0.01	0.16 ± 0.02	0.29 ± 0.08	-
2,4dtUra	0.23 ± 0.01	-	-	0.26 ± 0.05
2,4dtUrd	0.18 ± 0.01	-	-	0.20 ± 0.07

Supplementary References

1. Reichardt, C.; Crespo-Hernández, C. E., Room-temperature phosphorescence of the DNA monomer analogue 4-thiothymidine in aqueous solutions after UVA excitation. *J. Phys. Chem. Lett.* **2010,** *1* (15), 2239-2243.
2. Reichardt, C.; Crespo-Hernández, C. E., Ultrafast spin crossover in 4-thiothymidine in an ionic liquid. *Chem. Commun.* **2010,** *46* (32), 5963-5965.
3. Teles-Ferreira, D.; Conti, I.; Borrego-Varillas, R.; Nenov, A.; van Stokkum, I. H. M.; Ganzer, L.; Manzoni, C.; de Paula, A. M.; Cerullo, G.; Garavelli, M., A unified experimental/theoretical description of the ultrafast photophysics of single and double thionated uracils. *Chem. Eur. J.* **2019**, DOI: 10.1002/chem.201904541.
